# Mathematical modeling based on RT-qPCR analysis of SARS-CoV-2 in wastewater as a tool for epidemiology

**DOI:** 10.1038/s41598-021-98653-x

**Published:** 2021-09-30

**Authors:** Naďa Krivoňáková, Andrea Šoltýsová, Michal Tamáš, Zdenko Takáč, Ján Krahulec, Andrej Ficek, Miroslav Gál, Marián Gall, Miroslav Fehér, Anna Krivjanská, Ivana Horáková, Noemi Belišová, Paula Bímová, Andrea Butor Škulcová, Tomáš Mackuľak

**Affiliations:** 1 Department of Environmental Engineering, Faculty of Chemistry and Food Technology, Slovak University of Technology in Bratislava, Radlinského 9, 812 37 Bratislava, Slovak Republic; 2 Department of Molecular Biology, Faculty of Natural Sciences, Comenius University, Ilkovičova 6, 842 15 Bratislava, Slovakia; 3 Institute for Clinical and Translational Research, Biomedical Research Center, Slovak Academy of Sciences, Dúbravska Cesta 9, 84505 Bratislava, Slovakia; 4 Department of Inorganic Technology, Faculty of Chemical and Food Technology, Slovak University of Technology in Bratislava, Radlinského 9, 812 37 Bratislava, Slovakia; 5 Institute of Information Engineering, Automation, and Mathematics, Department of Mathematics, Faculty of Chemical and Food Technology, Slovak University of Technology in Bratislava, Radlinského 9, 812 37 Bratislava, Slovakia

**Keywords:** Molecular biology, Environmental sciences, Mathematics and computing

## Abstract

Coronavirus disease 2019 (COVID-19) pandemic caused by severe acute respiratory syndrome coronavirus 2 (SARS-CoV-2) emerges to scientific research and monitoring of wastewaters to predict the spread of the virus in the community. Our study investigated the COVID-19 disease in Bratislava, based on wastewater monitoring from September 2020 until March 2021. Samples were analyzed from two wastewater treatment plants of the city with reaching 0.6 million monitored inhabitants. Obtained results from the wastewater analysis suggest significant statistical dependence. High correlations between the number of viral particles in wastewater and the number of reported positive nasopharyngeal RT-qPCR tests of infected individuals with a time lag of 2 weeks/12 days (R^2^ = 83.78%/R^2^ = 52.65%) as well as with a reported number of death cases with a time lag of 4 weeks/27 days (R^2^ = 83.21%/R^2^ = 61.89%) was observed. The obtained results and subsequent mathematical modeling will serve in the future as an early warning system for the occurrence of a local site of infection and, at the same time, predict the load on the health system up to two weeks in advance.

## Introduction

**Figure 1:**
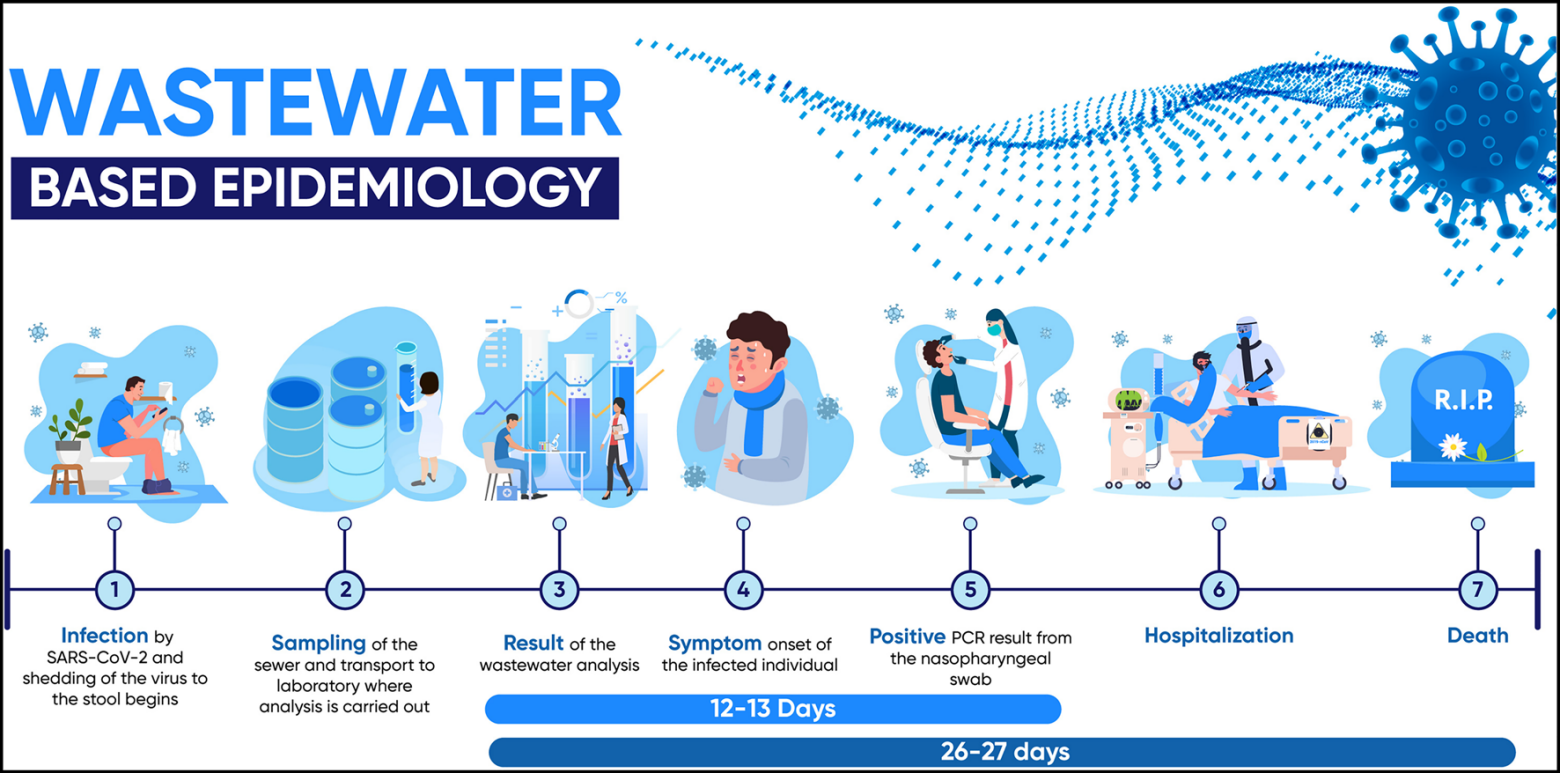
Visual presentation of wastewater based epidemiology. Increase in concentration of viral particles in the wastewater are considered to be around 12 days ahead before increase in clinical PCR testing and around 26 days before increase in deaths.

A thorough understanding of the current pandemic of COVID-19 is for public health officials a critical and ongoing challenge^1^. Currently, there are several epidemiological tools to gain control, however all of them have limitations. Rapid diagnostics tests are not accurate enough, the capacity of RT-qPCR testing may be insufficient, tracing contacts is based on personnel capacities and primarily symptomatic individuals are reported^1,2^. Long-term international experience with systematic monitoring of illegal drugs and their metabolites proposes that wastewater-based epidemiology (WBE) can be a highly effective way of surveillance for the presence of specific pathogens in communities^3–6^ (Fig. 1). Furthermore, it can be used to monitor the increasing or decreasing trend of the pathogen and its transmission^1,2^. The occurrence of hotspots caused by viruses may induce problems, especially in densely populated areas. Classical epidemiology lacks any prediction of highly infectious locations and is based mainly on clinical symptoms of infected individuals. Therefore in the case of COVID-19, where symptoms are delayed and many asymptomatic patients are observed, it has difficulties recognizing the acute onset of the disease^2^. SARS-CoV-2 or Severe Acute Respiratory Syndrome Coronavirus 2 causing respiratory illness COVID-19 is infectious diseases that resulted in 216 303 376 diagnosed cases and 4 498 451 deaths as of 30th August 2021 according to WHO^7^. Individuals infected by the virus exhibit various symptoms, but most often shortness of breath, dry cough and fever. The symptoms onset is usually within 2–14 days after exposure to the virus^8,9^. Nevertheless, around 45% of infected individuals show no symptoms of being asymptomatic throughout the course of the disease^10,11^.

At the beginning of 2020, several studies pointed out that viral particles of SARS-CoV-2 may be shed to the feces of infected individuals and therefore occur in the sewage system^12–14^. The assumption resides in the ability of SARS-CoV-2 to bind to angiotensin-converting enzyme 2 receptor (ACE2) that is abundant in the small intestine^15,16^. According to studies, viral particles are present in the stool early after infection and remain present during disease (9–16 days post symptom onset). Therefore they can be detected similarly as nasopharyngeal swabs in case of their negativity^17,18^. Based on these observations, WBE of SARS-CoV-2 may offer valuable additional and early information of infection tendency in a community^19^.

Wastewater-based monitoring of COVID-19 offers several advantages. Compared to clinical testing, it is a noninvasive cost-effective way of investigating virus transmission dynamics in entire communities. Moreover, there is a possibility of monitoring populations without access to standard healthcare systems. On the contrary, the downsides of the methods are a difficult analytical measurement of viral RNA, inconsistency in ongoing analyses with high starting costs^20^. Monitoring of SARS-CoV-2 can be further hampered by high portions of ballast waters and the chemical or biological composition of wastewater which may differ within the monitored region.

Our study describes the monitoring of RNA SARS-CoV-2 in wastewaters and defines a mathematical model of possible relations between RNA SARS-CoV-2 concentration in wastewater and subsequent increase of positive and death cases. Long-term monitoring was carried out in Bratislava, with an estimated population of 0.6 million inhabitants from September 2020 until March 2021.

## Materials and methods

### Characterization of investigated locations

**Figure 2:**
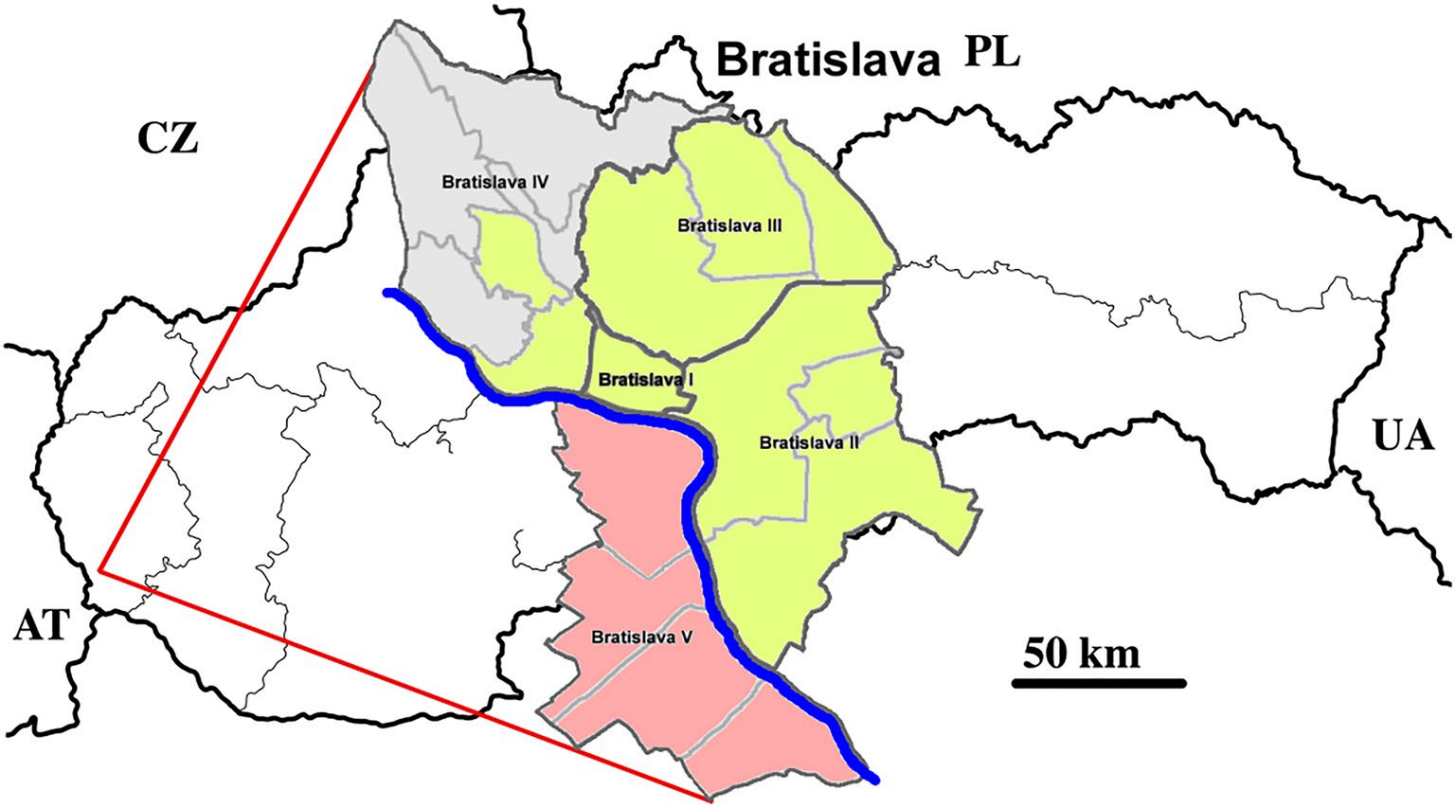
Geographical map of Slovakia and Bratislava with the districts. Green districts collect sewage to wastewater treatment plant (WWTP) Bratislava—Centrum (Vrakuna), represent 450,000 inhabitants. Red districts are areas containing sewage to WWTP Bratislava-Petrzalka representing 125,000 inhabitants. Map of Slovakia (https://sk.wikipedia.org/wiki/S%C3%BAbor:Slovakia_-_outline_map.svg) is licensed Creative Commons Attribution-ShareAlike 3.0 license. The license terms can be found on the following link: https://creativecommons.org/licenses/by-sa/3.0/. Map of Bratislava (https://sk.m.wikipedia.org/wiki/S%C3%BAbor:Bratislava_boroughs_outline_map.svg) is licensed Creative Commons Attribution-ShareAlike 4.0 license. The license terms can be found on the following link: https://creativecommons.org/licenses/by-sa/4.0/deed.cs (Edited by Tamas M, Inkscape, 1.0beta1, https://inkscape.org/).

Bratislava is a capital and the most populated city in the Slovak Republic, with approximately 600,000 inhabitants (Fig. 2). Petrzalka, with its satellites has a population of about 125,000 residents. It is the largest suburb of Bratislava (district Bratislava V) with the highest population density in Slovakia as well as all of Central Europe. It is located next to the city center on the right bank of the Danube River.

### Characterization of the wastewater treatment plants (WWTPs)

This study focused on the incidence of selected SARS-CoV-2 genes in two different Slovak (Bratislava) WWTPs. The first one is located in Bratislava—Centrum that treats almost all wastewater from the significant part of Bratislava. The second one is in Bratislava—Petrzalka that treats wastewater from Petrzalka (Table 1). WWTPs use a mechanical and biological stage (stage nitrification only); the produced sludge is digested and produced biogas energy is recovered.

**Table 1:** Basic characteristics of the WWTPs.

WWTP	Connected population (inhabitants)	Connected load (population equivalent)	Length of sewer (km)	A portion of industrial wastewater
Bratislava Centrum	450,000	450,000	1–30	Slightly (20%)
Bratislava Petrzalka	125,000	125,000	0.5–5	Very low (5%)

### Sampling

Automatic sampler devices were used to collect raw wastewater samples at the inflow point of WWTPs. Monitoring of wastewater was carried out at Bratislava—Centrum and Bratislava—Petrzalka from September 2020 until March 2021. During the sampling campaign more than 50 samples of influent were taken (Tables 1, 2).

**Table 2:**
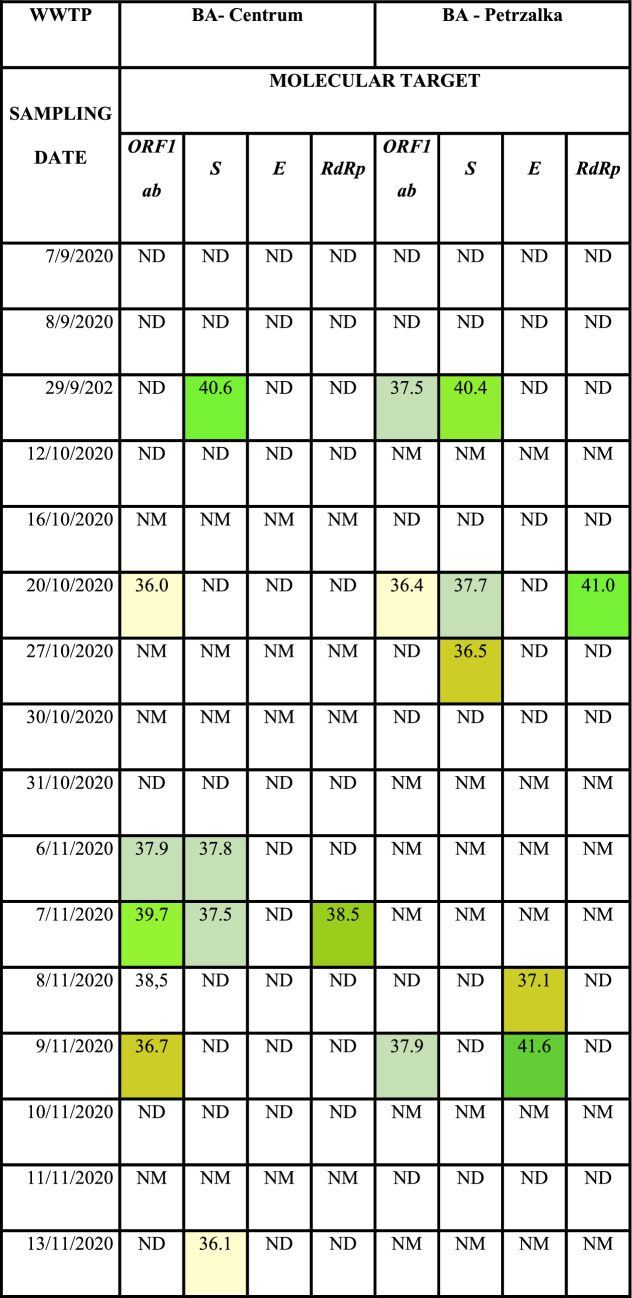

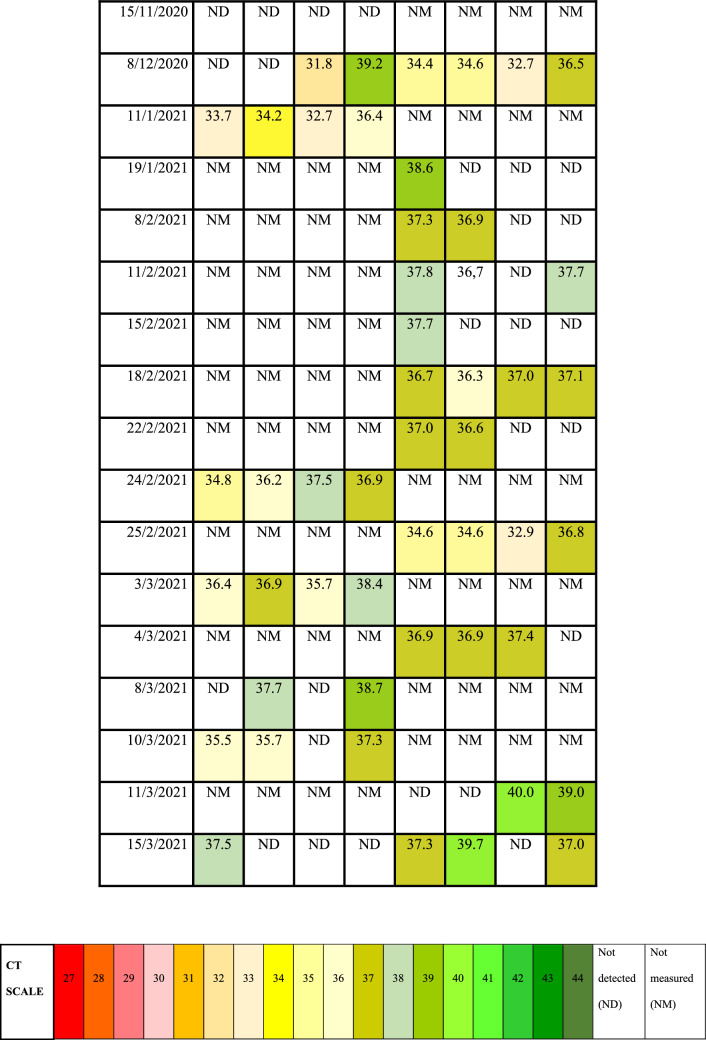
CT values measured by RT-qPCR of 4 genes (*ORF1ab, S, E, RdRp*) of influent at WWTPs. ND represents dates with performed measurement but no detected positivity of the samples. NM represents dates with not performed measurement of the samples.

Samples with a volume of 50 ml were taken every 15 min for a period of 24 h to obtain a daily representative sample (in total 4.8 l). The collected specimen was frozen within 2 h at − 20 °C and kept at this temperature until processing. This procedure follows a protocol established in several previous studies to prevent the material from biodegradation^21^.

### Sample processing

50 ml of wastewater was centrifuged at 4700 g for 30 min, and then 45 ml of clear supernatant was subjected to ultracentrifugation 125,893 g for 90 min. After ultracentrifugation, the pellet was resuspended in 1 ml of TRI reagent. RNA was isolated using Direct-zol RNA Miniprep (Zymo Research) according to the manufacturer’s instructions. Each sample was eluted in 100 µl of RNase-free water and RNA quantity was measured using NanoDrop ND-2000 (Nanodrop Inc.). A maximum of 8 µg of RNA per sample was concentrated by vacuum evaporation using Eppendorf Concentrator plus (Eppendorf).

### SARS-CoV-2 detection using quantitative RT-qPCR

The concentrated sample was resuspended in 11 µl of RNase-free water. A RevertAid First Strand cDNA synthesis kit (ThermoFisher Scientific) in a total volume of 20 μl was used for reverse transcription according to the manufacturer's instructions. 2 µl of the reverse transcription reaction product was analyzed in one RT-qPCR reaction using PerfeCTa qPCR FastMix II, low ROX (VWR) and specific primers for *E, RdRP* (described by WHO) and *ORF1ab, S* (described by Sigma Aldrich) genes (Supplementary Table S1). Initial denaturation was set to 10 min at 95 °C followed by 45 cycles of denaturation (95 °C, 30 s), annealing (59 °C, 30 s) and extension (72 °C, 30 s) using Stratagene Mx3005P (Agilent). Copies were estimated based on standard curves using synthetic SARS-CoV-2 RNA Control 1 (Australia/VIC01/2020, Genbank ID: MT007544.1).

### RT-qPCR product confirmation by Sanger sequencing

RT-qPCR products of each measured gene were further confirmed by Sanger sequencing. Out of all samples 8 were chosen for the confirmation. Sanger sequencing of the amplicons was carried by Microsynth Austria GmbH (Vienna, Austria). Quality control of sequences were assessed and confirmed in the BLAST standard database of nucleotide collection for coronavirus detection. Only highly similar sequences were reported. Reports from sequencing and BLAST results are accessible in Supplementary_material_2.

### Calculation of viral particles in sewage and mathematical modeling

Regression models (simple linear, Double Squared Root and Square root-Y logarithmic-X) have been used to describe the studied relations between the obtained data from wastewater and reported data related to the COVID-19 situation. The time-series analysis was used to compare the dependence between the wastewater time series and various time lags of the positive RT-qPCR tests (and Death cases) time series. To determine how well the time lags match up with the wastewater time series and in particular, at what point the best match (dependence with the highest R^2^) occurs, the cross-correlation function was applied. A generalized additive model (GAM) was used to illustrate the smoothed curves of relevant time series. The one-way ANOVA described the differences between the means of the positive RT-qPCR tests in particular days of the week based on the Studentized range statistic, Tukey's 'Honest Significant Difference' method.

The method's detection limit was calculated as the number of monitored inhabitants divided by minimal positive cases reported at the date with positive RT-qPCR wastewater analysis for SARS-CoV-2.

All statistical and mathematical operations were performed in the R environment and software program StatGraphics^22^.

## Results and discussion

Feces are the main contribution of SARS-CoV-2 presence in sewage, thus selected target genes were likewise detected by several research groups in feces or from rectal swabs^15,18,23^. Detection of several genes is vital as the RNA virus in wastewater is exposed to many environmental factors. Therefore, RNA may be strongly disintegrated resulting in poor amplification and variable detection of individual viral particles. We focused on detecting four genes *ORF1ab, S, E* and *RdRp* gene capturing different regions of the virus^24^. Similar target genes (*S, RdRP, ORF1ab*) were also selected by La Rossa et al.^25^. On the contrary, other researchers were investigating the presence of 3 different regions of nucleocapsid (*N*) gene in wastewater^26^. Currently, there is no consensus of targets recommended for detecting SARS-CoV-2 in sewage.

In our study 29 out of 52 analyzed wastewater samples (56%) were found to be positive. Table 2 displays CT values of detected genes in both WWTPs at given dates. First positive results were obtained on 29^th^ September with detected *S* gene at both WWTPs and *ORF1ab* gene at WWTP Bratislava-Petrzalka. In Bratislava—Centrum lowest CT detected was 31.8, whereas in Bratislava—Petrzalka, it was 32.7 on the same date. At least one of the targets was detected since this date at chosen WWTPs. The most often detected target was the *ORF1ab* gene with positivity of 33% (22 out of 66) from all positively detected targets.

Further, amplicons generated by RT-qPCR from 8 samples were confirmed by Sanger sequencing. This was done to prevent false negative results often obtained by RT-qPCR analysis as wastewater contains various RNA fragments and cross-reacting molecules^27^. 7 out of 8 passed quality control check and the amplicon sequences were aligned to coronavirus strain with 5 sequences confirming SARS-CoV-2 as first hits in the BLAST database (Supplementary table S3, Supplementary_material_2).

**Figure 3:**
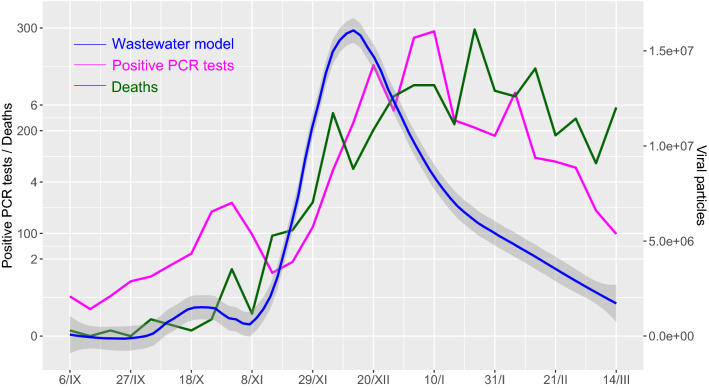
Reported numbers of positive RT-qPCR tests and reported numbers of death cases versus wastewater viral particles data model in Bratislava. The monitored time period was from 6^th^ September 2020 to 14th March 2021.

According to calibration curves, CTs presented in Table 2 were recalculated to the number of viral particles per milliliter. To calculate the daily loads of SARS-CoV-2 in wastewater, viral particles per ml were then multiplied by daily influents measured at the WWTPs (Supplementary Table S2). Then reported numbers of positive RT-qPCR tests and reported numbers of death cases were compared to the number of viral particles in wastewater in Bratislava (Fig. 3).

By interpolating the measured viral particles in the wastewater, we obtained a model useful for comparing the time series of positive RT-qPCR tests, death cases and viral particles (Fig. 3). Since the graph reveals apparent time shifts, we focused on the time lags between particular time series in our analysis.

The following Table 3 describes the dependencies between the time lags of reported positive RT-qPCR tests or death cases and the number of viral particles found on individual days in the wastewater of Bratislava.

**Table 3:** The values of R^2^ and time lags for dependence between the number of viral particles in wastewater and reported positive RT-qPCR tests and death cases.

	Daily time series				Weekly time series			
	Linear regression		Best regression model		Linear regression		Best regression model	
	Lag	R^2^	Lag	R^2^	Lag	R^2^	Lag	R^2^
Positive RT-qPCR	− 14	46.07%	− 12	52.65%	− 2	81.72%	− 2	83.78%
Deaths	− 7	28.23%	− 27	61.89%	− 4	48.06%	− 4	83.21%

It can be seen that the values of R^2^ for dependence between viral particles in wastewater and reported positive RT-qPCR tests and death cases (Table 3, Supplementary Fig. S2, S3) are better for the weekly time series than for the daily time series. This led us to focus our analysis on weekly time series. This decision is supported by the results of the ANOVA test where the significant difference among the means of the number of positive RT-qPCR tests in the particular days of the week was confirmed (p-value = 0.0054, F-value = 3.178, DF = 6, Supplementary Fig. S1). Therefore, we decided to use the time series of cumulative data with a 7 days period. The weekly time series of measured viral particles in wastewater reported numbers of positive RT-qPCR tests and reported numbers of death cases without/with the appropriate time lags are depicted in supplementary Fig. S4.

By analyzing the dependence between the weekly time series of measured viral particles in the wastewater and reported numbers of positive RT-qPCR tests with a time lag of 2 weeks, the models for estimation of the number of positive RT-qPCR tests were obtained. The results of fitting a linear (R^2^ = 81.72%, p-value < 0.0001, F-value = 116.2, DF = 26, Supplementary Fig. S5) and double square root (R^2^ = 83.78%, p-value < 0.0001, F-value = 134.3, DF = 26, Supplementary Fig. S6) models describe the relationship between the weekly time series of measured viral particles in the wastewater and reported numbers of positive RT-qPCR tests with the time lag of 2 weeks. The equations of the fitted models are:

Linear:

$$positive\,PCR\,lag2 = 76.578 + 0.0000135226 \cdot viral\,particles$$

Double square root:

$$positive\,PCR\,lag2 = {(7.23532+ 0.00233866 .\sqrt{viral\,particles})}^{2}$$

The models allow estimating the number of positive RT-qPCR tests with model fitting more than 80% (Figure S7).

By analyzing the dependence between the weekly time series of measured viral particles in the wastewater and reported numbers of death cases with a time lag of 4 weeks, the models for estimation of the number of death cases were also obtained. The results of fitting a linear (R^2^ = 48.06%, p-value < 0.0001, F-value = 24.06, DF = 26, Supplementary Fig. S8) and square root-Y logarithmic-X (R^2^ = 83.21%, p-value < 0.0001, F-value = 128.83, DF = 26, Supplementary Fig. S9) models describe the relationship between the weekly time series of measured viral particles in the wastewater and reported numbers of death cases with the time lag of 4 weeks. The values of R^2^ show that the non-linear model is significantly better than the linear. The equations of the fitted models are:

Linear:

$$deaths\,lag4 = 2.71168 + 0.000000326376 \cdot viral\,particles$$

Square root-Y logarithmic-X:

$$deaths\,lag4 = {(-1.75359 + 0.26318 \cdot ln (viral\,particles))}^{2}$$

The non-linear model allows estimating the number of death cases with model fitting more than 80% (Supplementary Fig. S10).

Further, we looked at the detection limit of wastewater monitoring related to the number of positive RT-qPCR cases within a 12 days shift. Individually, the detection limit of WWTP Bratislava—Vrakuna is 1 RT-qPCR positive case per 25,000 people on 15/3/2021, while Bratislava—Petrzalka reached 1 per 4,808 on 19/1/2021. The results are also limited by reporting the positive RT-qPCR cases as separate data for each city district has been available since 10/11/2020. For Bratislava the detection limit was 1 per 8,099 on 29/9/2020 when both WWTPs were combined. It is important to stress that the results are related and strongly biased to reported RT-qPCR positive cases which vary between the countries, their testing capacities and contact tracing. In comparison, the study by Ahmed et al., could not detect SARS-CoV-2 in wastewater until 100 reported cases per 100,000 people. On the contrary, Medema et al., was capable of detecting SARS-CoV-2 in the wastewater before the first confirmed results in the monitored area^28,29^. Another reason for such contrasting results is probably the different quality of the wastewater and various methodological approach influencing the detection limit. Each sewer has unique properties such as temperature, pH, presence of chemical substances and biological composition. All of these factors play a role in the degradation of viral particles. Therefore investigation of these properties is crucial and each methodological approach to detect SARS-CoV-2 from wastewater is unique for a given sewer system. However, some unification of the methodology would be vital for future comparison of the results.

Until now there was no confirmed case of infection from wastewater thus oral-fecal route is unlikely. The reason may be the inactivation of SARS-CoV-2 by gastrointestinal fluids or an unfavorable wastewater environment^16,30,31^. The amount of the virus shed to the feces may vary in time as well as between the patients^23^. Once the virus or its particles enter the sewage system, it is diluted by other types of water (industrial, rainfalls, water from snow melting, etc.) and is exposed to various physical and chemical factors. So far, viral RNA appears to be stable until reaching the primary settling tank of WWTP. Compared to non-enveloped viruses, SARS-CoV-2 has an affinity to wastewater solids, so a portion of the virus is probably sorbed on sewage walls and later to primary sludge^30,32^. On the contrary, wastewater surveillance offers several benefits once we have a working monitoring system. In September 2020, Larsen and Wigginton published in Nature Biotechnology that theoretically, wastewater surveillance can be in 7 days lead before rapid diagnostics tests and 13–15 days before delayed diagnostic tests^1,33^. This is in agreement with our observations where results from wastewater dated to the day of sampling were in 14 days (2 weeks) lead before standard clinical RT-qPCR testing. If we add 1–2 days of sample pretreatment and analysis we are reaching for 12–13 days advance before reported positive RT-qPCR tests. Novelty in the presented study is that a similar correlation can be found with deaths reported in 28 days (4 weeks) after the detected increase of viral particles in the wastewater. Although we can not specify the number of infected persons, observation of trends in near real-time can help us understand community transmission. In communities with low capacity of clinical testing and delays of diagnostics wastewater surveillance can be a temporary solution. Moreover, as it is relatively cost-effective and less invasive, wastewater monitoring can be used in low-income countries^34,35^. However in communities with working traditional testing, data obtained from wastewater are only additional information in controlling pandemics. They can be used to check the reliability of the introduction of novel technologies or protocols.

For successful monitoring of SARS-CoV-2 in the wastewater on a larger scale, public authorities must realize the importance of possible scalability of the method^36^. National agencies should show interest and provide financial and material support for research teams and water companies in the form of grants. Monitoring of COVID-19 and its mutations will require modification of the methods when compared to the monitoring of drugs or metabolites^36^. Optimistic presumption for successful cooperation is the fact that across Europe there is a working consortium of institutes and universities focusing on monitoring illegal drugs in the wastewaters^37,38^. European Commission at the beginning of March created the HERA incubator. One of the focuses is the systematic surveillance of the wastewaters, including genetic sequencing at WWTPs with a connected population of over 150,000 individuals^39^.

In the presented study, we detected RNA SARS-CoV-2 in wastewaters and displayed mathematical correlations between tested wastewater samples, positive RT-qPCR tests and death cases in Bratislava, Slovakia. The obtained results and subsequent mathematical modeling will be able to serve in the future as an early warning system for the occurrence of a local site of infection and at the same time will allow to predict the load on the health system up to two weeks in advance. Because each wastewater has its own characteristics (pH, temperature, (bio)chemical composition, etc.), it is necessary to approach this in other monitored localities when taking and processing samples, evaluating the results and creating the appropriate mathematical model.

## Supplementary Information

Supplementary Information 1.

Supplementary Information 2.
