# Supplementary material for: Mathematical modeling based on RT-qPCR analysis of SARS-CoV-2 in wastewater as a tool for epidemiology
Source: Sci Rep. 2021 Sep 30;11:19456. doi: 10.1038/s41598-021-98653-x (PMC8484274; doi:10.1038/s41598-021-98653-x)
Supplement: Supplementary file 1 — Supplementary Information 1. [file 41598_2021_98653_MOESM1_ESM.docx]

**Mathematical modeling based on RT-qPCR analysis of SARS-CoV-2 in wastewater as a tool for epidemiology**

Naďa Krivoňáková^5,✝^, Andrea Soltysová^2,3,✝^, Michal Tamáš^1,✝^, Zdenko Takáč^5,✝^, Ján Krahulec^2^ , Andrej Ficek^2^, Miroslav Gál^4^, Marián Gall^5^, Miroslav Feher^1^, Anna Krivjanská^1^, Ivana Horáková^1^, Noemi Belišová^1^, Paula Bímová^1^, Andrea Butor Škulcová^1^, Tomáš Mackuľak^1*^

**Supplementary material**

**Supplementary Table S1:** Specific primers and probes used for RT-qPCR analysis of RNA SARS-CoV-2

| Name | Sequence |
| --- | --- |
| 2019-nCoVAssay3Forward | CCGCAAGGTTCTTCTTCGTAAG |
| 2019-nCoVAssayReverse | TGCTATGTTTAGTGTTCCAGTTTTC |
| 2019-nCoVDLProbeA(3&4) | [6FAM]AAGGATCAGTGCCAAGCTCGTCGCC[BHQ1] |
| 2019-nCoVAssay5Forward | CAGGTATATGCGCTAGTTATCAGAC |
| 2019-nCoVAssay5Reverse | CCAAGTGACATAGTGTAGGCAATG |
| 2019-nCoVDLProbe(5&6) | [6FAM]AGACTAATTCTCCTCGGCGGGCACG[BHQ1] |
| E_Sarbeco_F1 | ACAGGTACGTTAATAGTTAATAGCGT |
| E_Sarbeco_R2 | ATATTGCAGCAGTACGCACACA |
| E_Sarbeco_P1 | [FAM]ACACTAGCCATCCTTACTGCGCTTCG[BHQ1] |
| RdRP_SARSr-F2 | GTGARATGGTCATGTGTGGCGG |
| RdRP_SARSr-R1 | CARATGTTAAASACACTATTAGCATA |
| RdRP_SARSr-P2 | [FAM]CAGGTGGAACCTCATCAGGAGATGC[BHQ1] |

**Supplementary Table S2:** Daily influents and viral load per day at WWTPs Bratislava - Centrum and Bratislava - Petrzalka on sampling dates. To minimize error, the values in bold were calculated as half of the minimal value of the detected values in the particular WWTP. NM (not measured) represent dates with no performed analysis.

|  | Daily influents at WWTP (m^3^/day) | | Viral load per day (copies) | |
| --- | --- | --- | --- | --- |
| Date: | Bratislava - Centrum | Bratislava - Petrzalka | Bratislava - Centrum | Bratislava - Petrzalka |
| 7/9/2020 | 147520 | 41374 | **4,73E+06** | **1,44E+07** |
| 8/9/2020 | 80250 | 20322 | **4,73E+06** | **1,44E+07** |
| 29/9/202 | 88600 | 21155 | 9,45E+06 | 2,88E+07 |
| 12/10/2020 | 226810 | 35608 | **4,73E+06** | NM |
| 16/10/2020 | 193970 | 38264 | NM | **1,44E+07** |
| 20/10/2020 | 108130 | 23824 | 3,70E+09 | 1,86E+09 |
| 27/10/2020 | 89520 | 22757 | NM | 1,12E+09 |
| 30/10/2020 | 231050 | 41413 | NM | **1,44E+07** |
| 31/10/2020 | 138750 | 26960 | **4,73E+06** | NM |
| 6/11/2020 | 94570 | 23492 | 7,17E+08 | NM |
| 7/11/2020 | 90940 | 21220 | 2,75E+09 | NM |
| 8/11/2020 | 91570 | 23718 | 6,63E+08 | 1,10E+09 |
| 9/11/2020 | 93820 | 23173 | 3,29E+09 | 4,94E+08 |
| 10/11/2020 | 91110 | 23029 | **4,73E+06** | NM |
| 11/11/2020 | 88490 | 23042 | NM | **1,44E+07** |
| 13/11/2020 | 86420 | 22256 | 2,83E+09 | NM |
| 15/11/2020 | 94530 | 23857 | **4,73E+06** | NM |
| 8/12/2020 | 81540 | 20923 | 1,93E+10 | 1,22E+10 |
| 11/1/2021 | 85 000 | NM | 9,42E+09 | NM |
| 19/1/2021 | NM | 21 194 | NM | 2,63E+08 |
| 8/2/2021 | NM | 32 030 | NM | 1,05E+09 |
| 11/2/2021 | NM | 25 606 | NM | 2,34E+09 |
| 15/2/2021 | NM | 26 250 | NM | 5,65E+08 |
| 18/2/2021 | NM | 25 296 | NM | 2,63E+09 |
| 22/2/2021 | NM | 24 883 | NM | 9,69E+08 |
| 24/2/2021 | 96 910 | NM | 3,55E+09 | NM |
| 25/2/2021 | NM | 23 206 | NM | 6,06E+09 |
| 3/3/2021 | 91 120 | NM | 1,92E+09 | NM |
| 4/3/2021 | NM | 21 680 | NM | 7,37E+08 |
| 8/3/2021 | 87 990 | NM | 2,61E+09 | NM |
| 10/3/2021 | 86 040 | NM | 3,37E+09 | NM |
| 11/3/2021 | NM | 22 075 | NM | 1,55E+09 |
| 15/3/2021 | 86 460 | 20 884 | 5,82E+08 | 2,29E+09 |

**Supplementary table S3:** Sequencing results of 8 randomly chosen samples for confirmation of amplicons obtained from RT-qPCR. Table describing given gene, sample, quality control from sequencing, E-value calculated and description provided by BLAST.

| **Gene** | **Sample** | **Quality control** | **E-value** | **Description** |
| --- | --- | --- | --- | --- |
| P3 | 24/02/2021 Vrakuna | Moderate | 3E-34 | Coronavirus SARS-CoV-2 |
| P3 | 11/02/2021 Petrzalka | Passed | 1E-37 | Coronavirus SARS-CoV-2 |
| P5 | 24/02/2021 Vrakuna | Moderate | 2E-07 | Coronavirus SARS-CoV-2 |
| P5 | 11/02/2021 Petrzalka | Moderate | - | Not aligned |
| E | 18/02/2021 Petrzalka | Passed | 1E-14 | Coronavirus SARS-CoV-2 |
| E | 03/03/2021 Vrakuna | Passed | 1E-12 | Coronavirus Pangolin |
| RdRP | 11/02/2021 Petrzalka | Passed | 1E-07 | Bat Coronavirus |
| RdRP | 03/03/2021 Vrakuna | Moderate | 4E-07 | Bat Coronavirus |

**
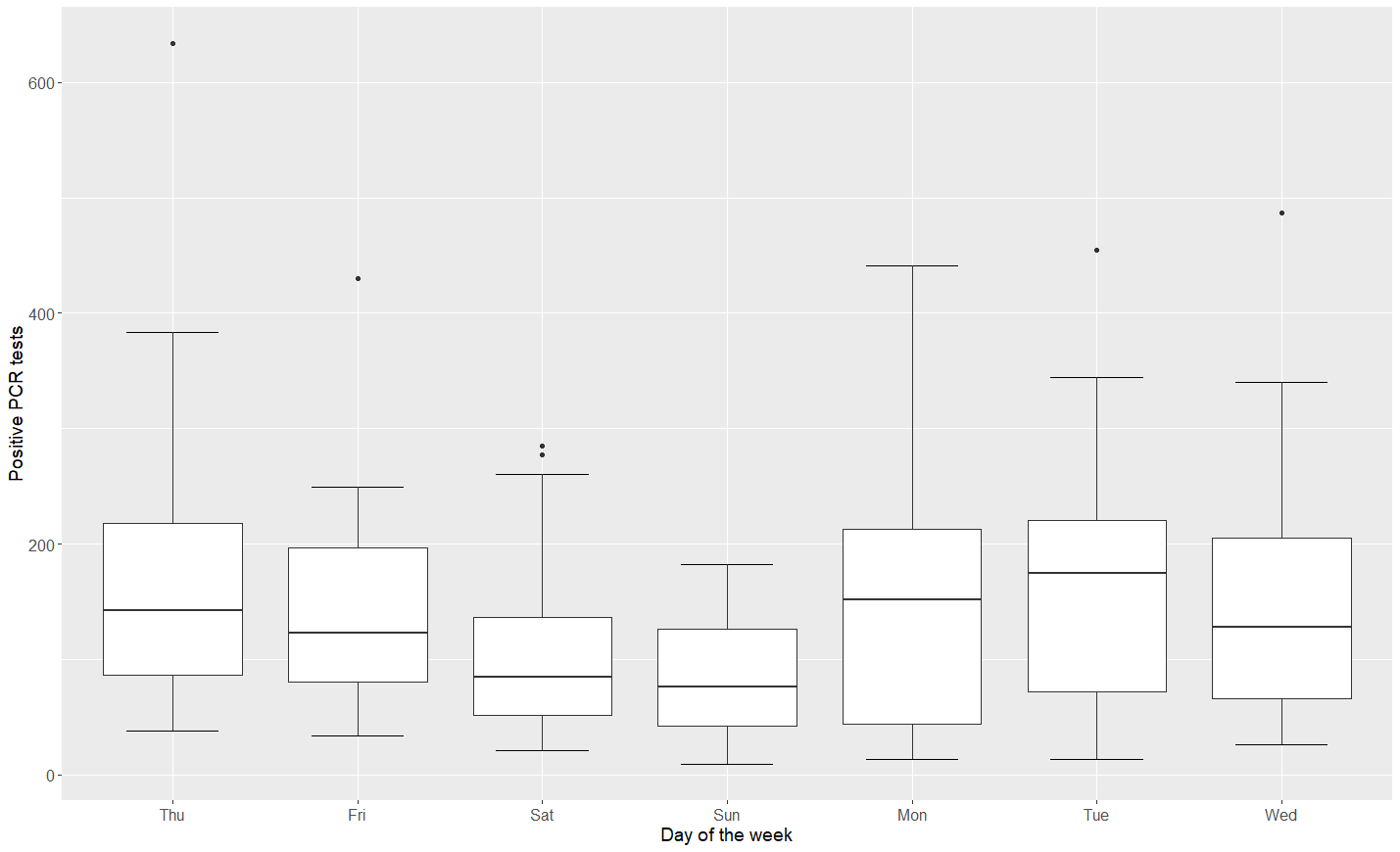
**

**Supplementary Figure S1:** Daily mean flow box plots of positive RT-qPCR tests in Bratislava showed a clear difference among days of the week, where weekends evidently differ from weekdays. It was verified by ANOVA test which revealed significant differences among the daily means (p-value=0.0054, F-value=3.178, DF=6). Levene’s test confirmed the homogeneity of variances (p-value=0.05479, F-value=2.1026, DF=6).

**
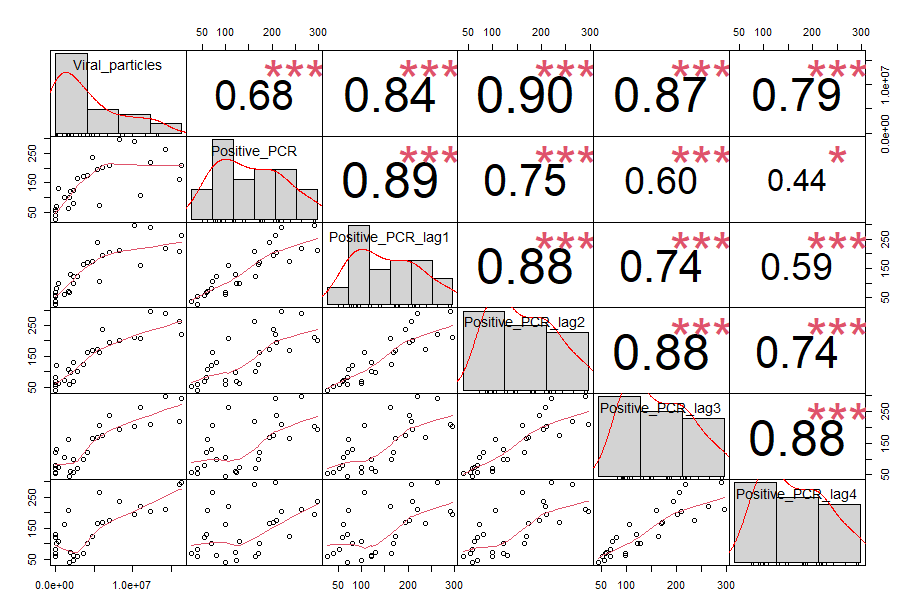
**

**Supplementary Figure S2:** Correlation matrix of the following six variables: the number of viral particles, the number of positive RT-qPCR tests and the number of positive RT-qPCR tests with the time lags of 1, 2, 3 and 4 weeks. The first row of the matrix shows that for the number of viral particles there is the most significant correlation (correlation coefficient equal to 0.90) with the number of positive RT-qPCR tests with a time lag of 2 weeks.

**
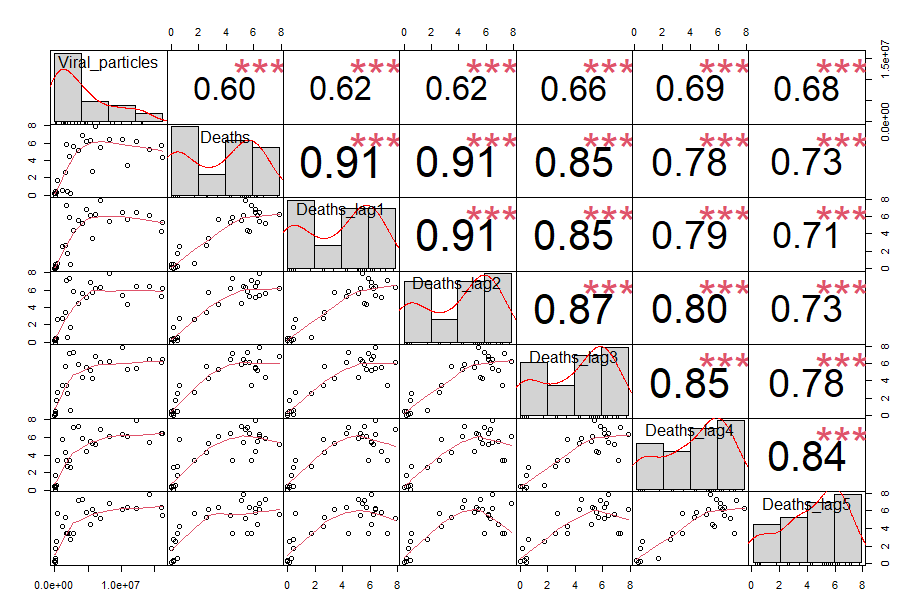
**

**Supplementary Figure S3:** Correlation matrix of the following seven variables: the number of viral particles, the number of deaths and the number of deaths with the time lags of 1, 2, 3, 4 and 5 weeks. The first row of the matrix shows that for the number of viral particles there is the most significant correlation (correlation coefficient equal to 0.69) with the number of deaths with the time lag of 4 weeks.

**
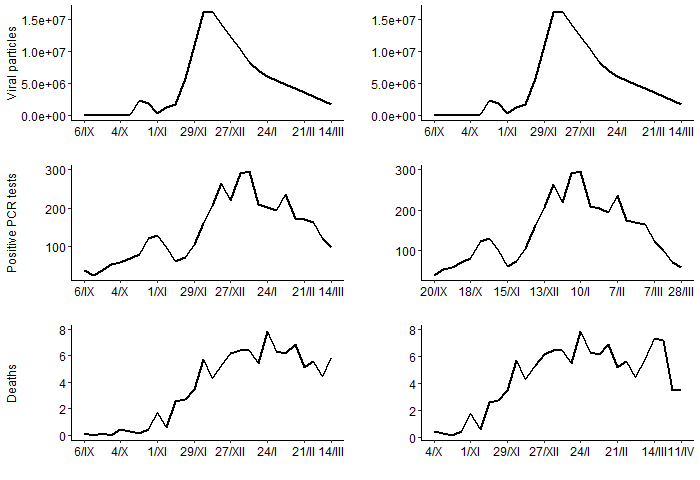
**

**Supplementary Figure S4:** The time series (from September 2020 to March 2021) of measured viral particles (linearly interpolated) in the first line, reported numbers of positive RT-qPCR tests in the second line (with no time lag on the right and the time lag of two weeks on the left) and reported numbers of deaths (with no time lag on the right and time lag of four weeks on the left).

**
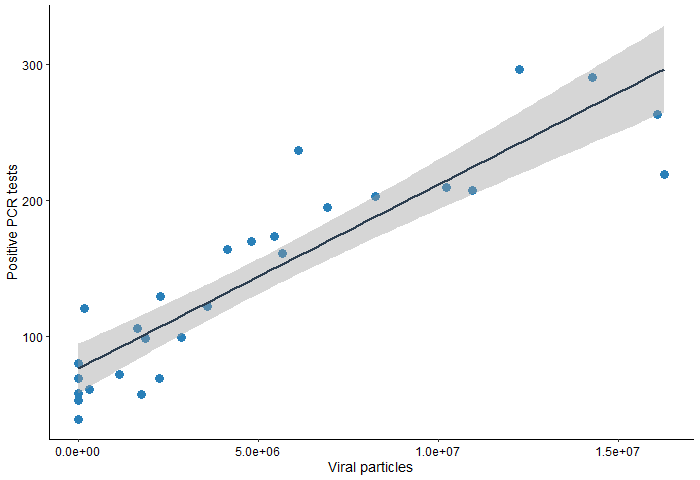
**

**Supplementary Figure S5:** The linear dependence between the measured viral particles in the wastewater and reported numbers of positive RT-qPCR tests with the time lag of 2 weeks, both time series considering cumulative data with 7 days period. Linear regression (Correlation Coefficient = 0.903986, R^2^=81.72%, F-value=116.2, DF=26):

$positive PCR lag2 = 76.578 + 0.0000135226 \cdot viral particles$.

**a)**

**
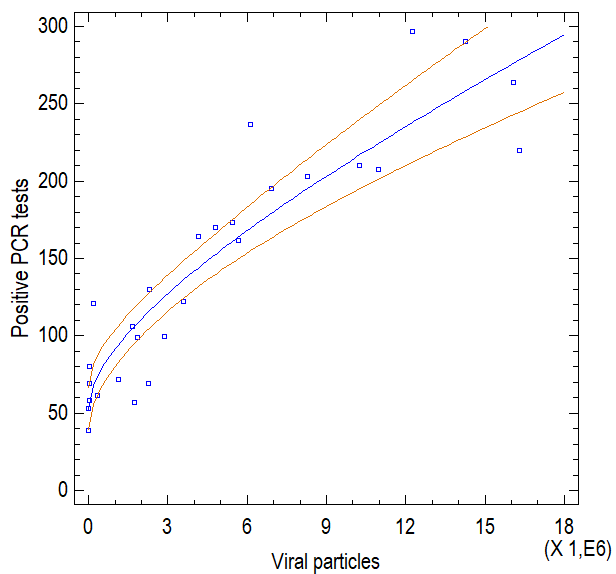
**

**b)**

**
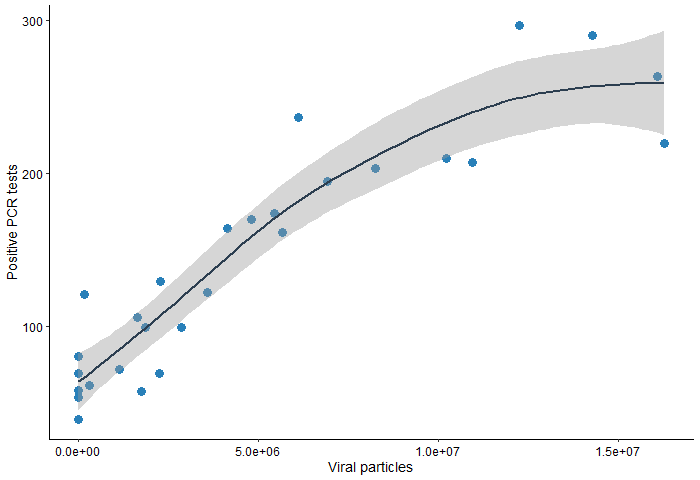
**

**Supplementary Figure S6:** The non-linear dependence between the measured viral particles in the wastewater and reported numbers of positive RT-qPCR tests with the time lag of 2 weeks, both time series considering cumulative data with 7 days period.

a) Double Squared Root model (R^2^=83.78%, F-value=134.3, DF=26, p-value<0.0001):

$positive PCR lag2 = (7.23532 + 0.00233866 \cdot\sqrt{viral particles}){}^{2}$.

b) The curve of the same non-linear dependence smoothed by the GAM method.

**a)**

**
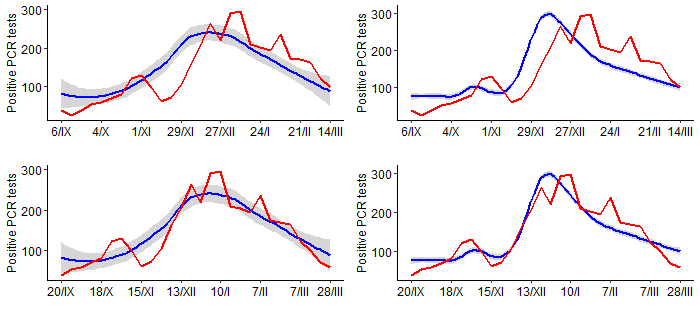
**

**b)**

**
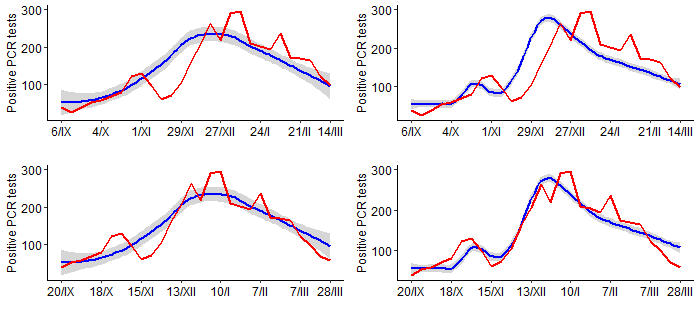
**

**Supplementary Figure S7:** Reported numbers of positive RT-qPCR tests (with no time lag in the first row and with the time lag of 2 weeks in the second row) vs a) the linear wastewater model / b) the best (among considered) wastewater model estimation of positive RT-qPCR tests (smoothed with span=0.75 on the left and span=0.25 on the right). The monitored time period was from September 2020 to March 2021.

**
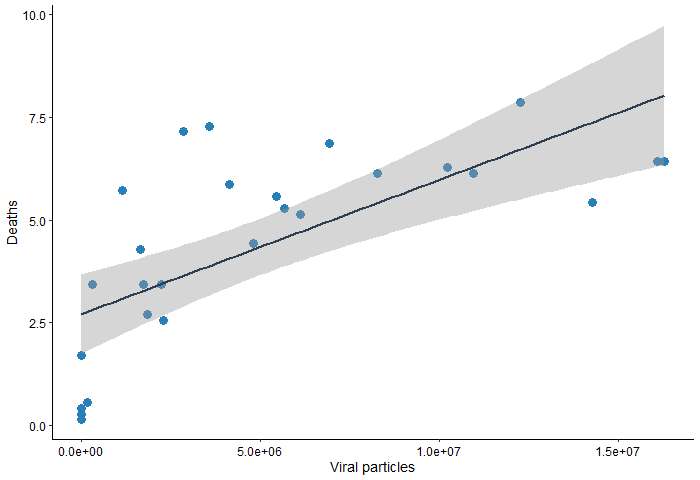
**

**Supplementary Figure S8:** The linear dependence between the measured viral particles in the wastewater and reported numbers death cases tests with the time lag of 4 weeks, both variables time series considering cumulative data with 7 days period. Linear regression (Correlation Coefficient = 0.693253, R^2^=48.06%, F-value=24.06, DF=26):

$deaths lag4 = 2.71168 + 0.000000326376 \cdot viral particles$.

**a)**

**
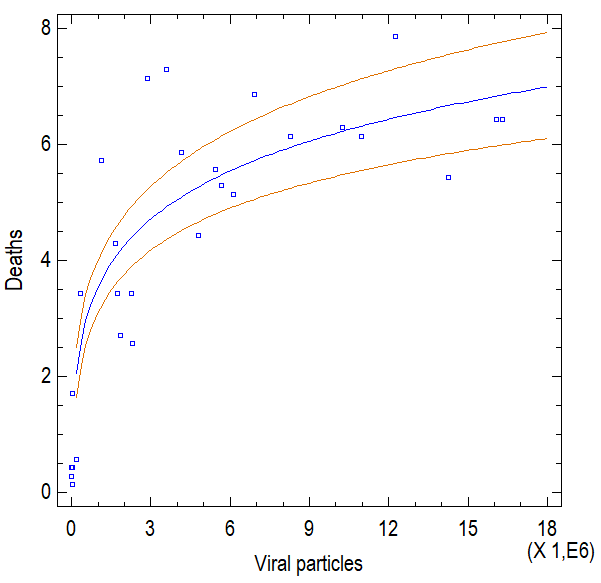
**

**b)**

**
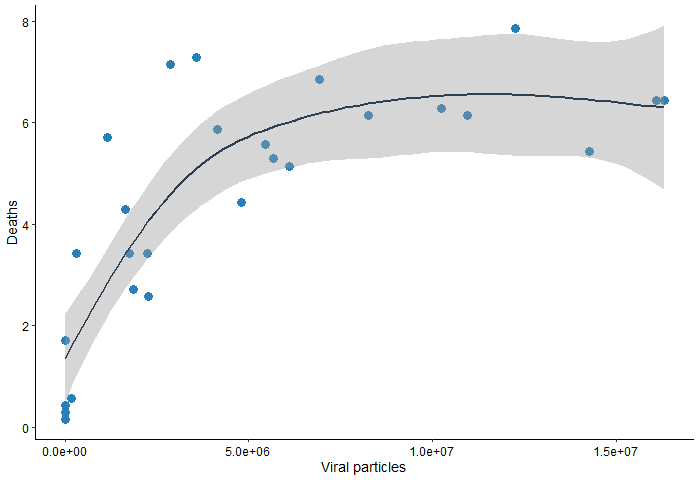
**

**Supplementary Figure S9:** The non-linear dependence between the measured viral particles in the wastewater and reported numbers of deaths with the time lag of 4 weeks, both time series considering cumulative data with 7 days period. a) Square root-Y logarithmic-X (R^2^=83.21%, p-value<0.0001, F-value=128.83, DF=26):

$$deaths lag4 = (-1.75359 + 0.26318 \cdot ln (viral particles)){}^{2}$$

b) The curve of the same non-linear dependence smoothed by the GAM method.

**a)**

**
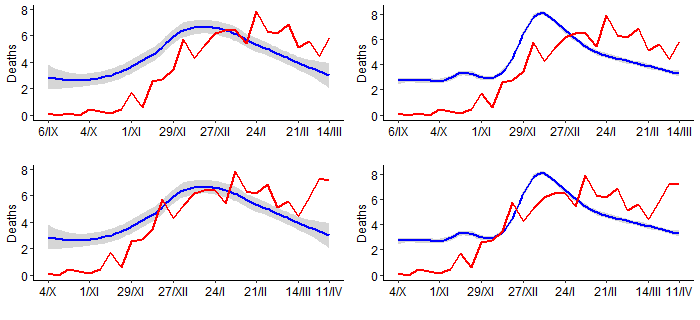
**

**b)**

**
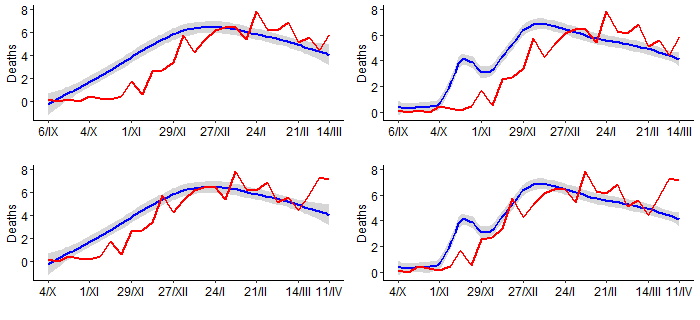
**

**Supplementary Figure S10:** Reported numbers of death cases (with no time lag in the first row and with the time lag of 4 weeks in the second row) vs a) the linear wastewater model / b) the best (among considered) wastewater model estimation of death cases (smoothed with span=0.75 on the left and span=0.25 on the right). The monitored time period was from September 2020 to March 2021.
